# Supplementary material for: Bioinspired enzymatic compartments constructed by spatiotemporally confined in situ self-assembly of catalytic peptide
Source: Commun Chem. 2022 Jul 9;5:81. doi: 10.1038/s42004-022-00700-9 (PMC9814850; doi:10.1038/s42004-022-00700-9)
Supplement: Supplementary file 2 — Supplementary Information [file 42004_2022_700_MOESM2_ESM.pdf]

Supplementary Information

Bioinspired Enzymatic Compartments Constructed by Spatiotemporally Confined *in situ* Self-Assembly of Catalytic Peptide

Yaling Wang,[1,+] Tiezheng Pan,[2,+] Xuewen Wei,[1] Fangcui Su,[3] Ang Li,[1] Yifan Tai,[1] Tingting Wei,[1] Qian Zhang,[1] Deling Kong,[1,4,*] Chunqiu Zhang[1,*]

[1]State Key Laboratory of Medicinal Chemical Biology, Key Laboratory of Bioactive Materials of Ministry of Education and College of Life Sciences, Nankai University, Tianjin, China

[2]School of Life Sciences, Northwestern Polytechnical University, Xi'an, China

[3]Key Laboratory for Molecular Enzymology and Engineering of Ministry of Education, School of Life Sciences, Jilin University, China

[4]Frontiers Science Center for Cell Responses, Nankai University, Tianjin, China

Email: zhangcq@nankai.edu.cn; kongdeling@nankai.edu.cn

+These authors contributed equally.

Supplementary Methods

Transmission electron microscopy Imaging (TEM)

The GPs, TPE-Q18H@GPs solutions were adsorbed onto carbon-coated, 300-mesh copper grids (Zhongjingkeyi Technology Co. Ltd., Beijing, China; glow-discharged before use) for 2 min and excess solution was removed with the filter paper. After drying, TEM micrographs were recorded on an FEI Talos L120C G2 transmission electron microscope at 120 kV acceleration voltage.

The peptide assemblies were prepared from 168 μM TPE-Q18H solutions (1× PBS, pH=7.4), followed by the adsorption onto ultrathin carbon-coated, 300-mesh copper grids (Zhongjingkeyi Technology Co. Ltd., Beijing, China; glow-discharged before use) for 2 min, and excess solution was removed with the filter paper. Then, 1 % (w/v) uranyl acetate was added and incubated for 1 min, followed by the removal of excess solution. After drying, TEM micrographs were recorded on a Talos F200X G2 transmission electron microscope at 120 kV acceleration voltage.

Atomic force microscope imaging (AFM)

The peptide assemblies were prepared from 178.24 μM TPE-Q18H solutions (1× PBS, pH=7.4). 10 μL droplets of the peptide solution was dropped onto clean mica slides and kept at room temperature for drying. Slides were then washed briefly with Milli-Q water and excess solution was removed with the filter paper. After drying, AFM micrographs were recorded on a BRUKER Dimension Icon atomic force microscopy.

Field emission scanning electron microscope (FE-SEM)

The GPs dry power was coated on silicon slide (adhered on the plat by conductive adhesive) or conductive adhesive, then sputtered for 30 s with gold. SEM micrographs were recorded on a JSM-7900F scanning electron microscope at 1.0 kV acceleration voltage.

Gas adsorption

The low-pressure nitrogen adsorption and desorption isotherms were measured at 77 K using a Micromeritics ASAP 2460 instrument. Brunauer-Emmett-Teller (BET) method was used to calculate specific surface areas based on the adsorption data. Approximately 50 mg sample was used. The sample was outgassed at 80 °C for 10 h before the measurements.

Confocal laser scanning microscope (CLSM)

TPE-Q18H@GPs and TPE-Q18H@DTAF-GPs solutions were dropped onto glass slides, and then sealed with cover glasses for observation. Images were observed and captured using a Leica CLSM 800 laser scanning confocal microscope (Leica, Germany).

Fluorescence spectroscopy

Fluorescence spectra of TPE were obtained on a fluorescence spectrophotometer (HITACHI F-7000). TPE-Q18H or TPE-Q18H@GPs were added into cuvettes, and the emission spectrum of TPE was recorded with the excitation at 365 nm with 5 nm excitation and 2.5 nm emission slit.

Fluorescence spectra of thioflavin-T were measured on a fluorescence spectrophotometer (HITACHI F-7000), and thioflavin-T was added to the final concentration of 100 μM and incubated with TPE-Q18H or TPE-Q18H@GPs for ten minutes. The excitation wavelength was set to be 440 nm with 10 nm excitation and 5 nm emission slit.

For monitoring the salt responsive assembly process of TPE-Q18H and TPE-Q18H@GPs, Th T (final concentration of 250 μM) was incubated with TPE-Q18H or TPE-Q18H@GPs in water. After ten minutes, appropriate 10× PBS was added to the final concentration of 1× PBS to trigger the assembly. The fluorescence intensity of Th T at 488nm was monitored by fluorescence spectrophotometer (Lengguang F97 Pro) before and after adding salt. For TPE-Q18H@GPs

groups, stirring the solution every one minute to prevent the sedimentation of particles. The excitation wavelength was set to be 440 nm with 10 nm excitation and 5 nm emission slit.

Supplementary Figures

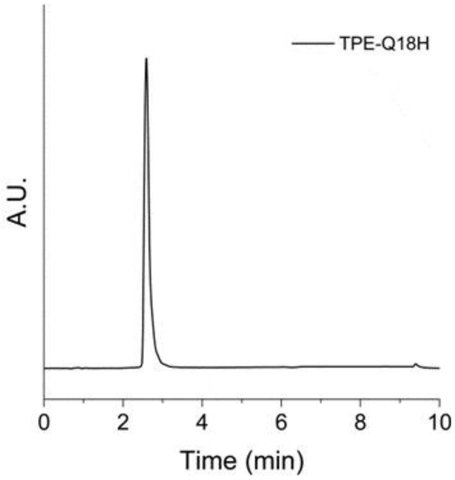

Supplementary Figure 1. Analytical HPLC of synthesized TPE-Q18H.

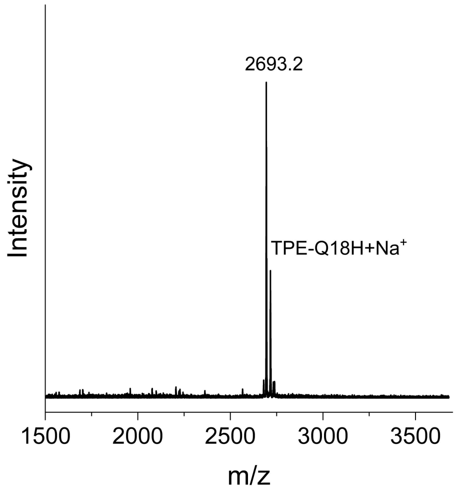

Supplementary Figure 2. MALDI-TOF MS spectra analysis of synthesized TPE-Q18H.

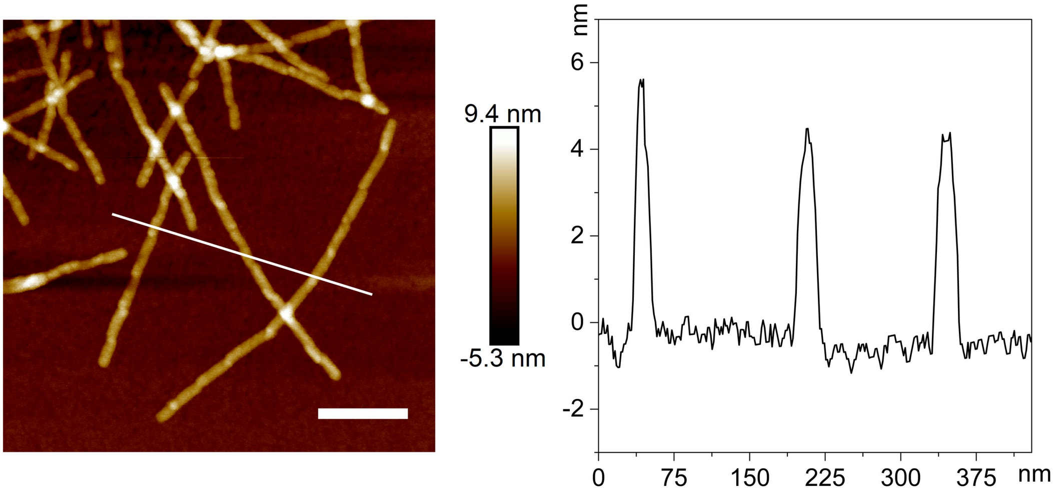

Supplementary Figure 3. AFM image of TPE-Q18H nanofibers and height profile along the white line. Scale bar: 150 nm

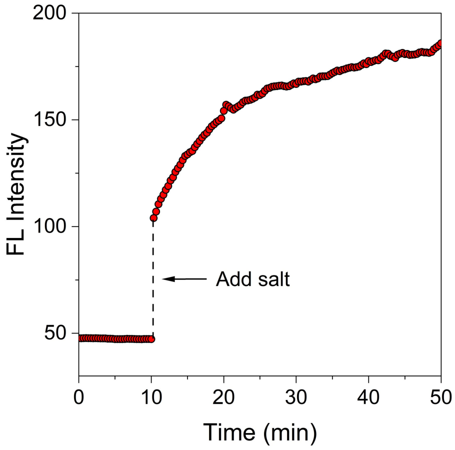

Supplementary Figure 4. The fluorescence intensity of Th T incubated with TPE-Q18H before and after adding salt at 488 nm. λ_{ex} =440 nm. Th T: 250 μM.

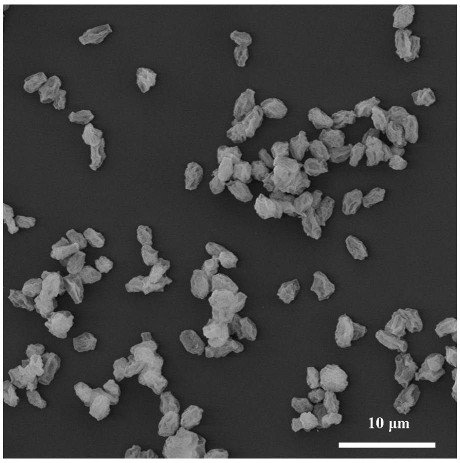

Supplementary Figure 5. SEM image of GPs. Scale bar: 10 μm

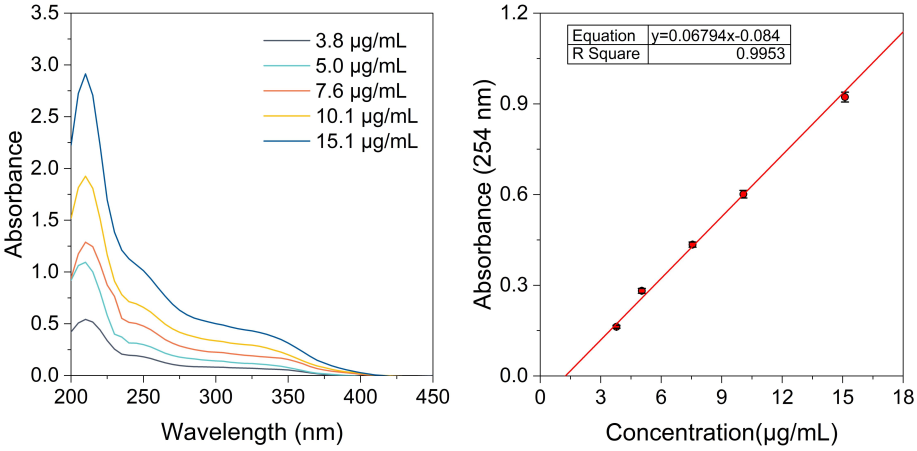

Supplementary Figure 6. UV-vis spectra of various concentrations of TPE-Q18H in 1×PBS and standard curve of TPE-Q18H at 254 nm. Path length= 1.0 mm. Data are presented as the mean ± SD (dot with error bar) of n = 3 independent measurements.

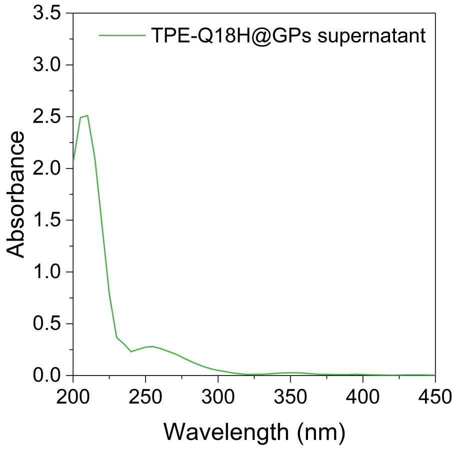

Supplementary Figure 7. UV-vis spectra of TPE-Q18H@GPs supernatant. Path length= 1.0 mm. The absorbance at 254 nm is 0.28 and the concentration is 5.36 μg/mL and the encapsulation efficiency is 98.23%.

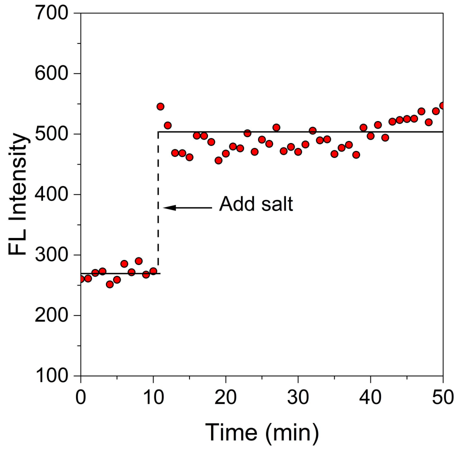

Supplementary Figure 8. The fluorescence intensity of Th T incubated with TPE-Q18H@GPs before and after adding salt at 488 nm. λ_{ex} =440 nm. Th T: 250 μM.

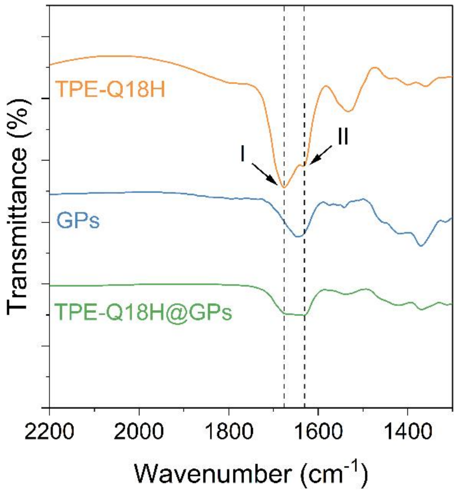

Supplementary Figure 9. FTIR spectra of assembled TPE-Q18H, empty GPs, and TPE-Q18H @GPs.

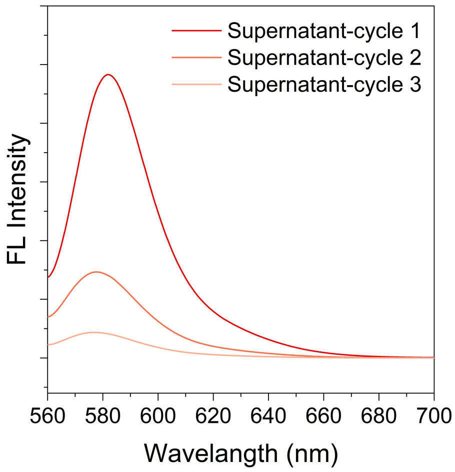

Supplementary Figure 10. Fluorescence spectra of supernatants of Rhodamine@GPs during three times centrifugations. λ_{ex}=540 nm.

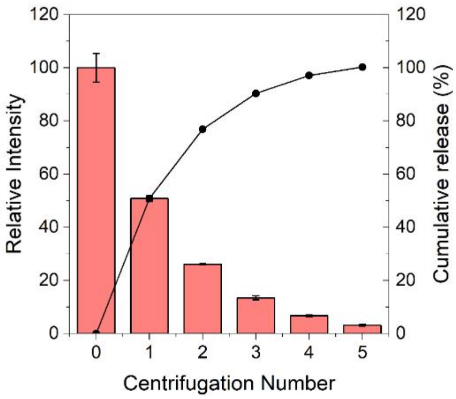

Supplementary Figure 11. The releasing kinetics of Rhodamine from GPs during centrifugations. Data are presented as the mean ± SD (column with error bar) of n = 3 independent measurements.

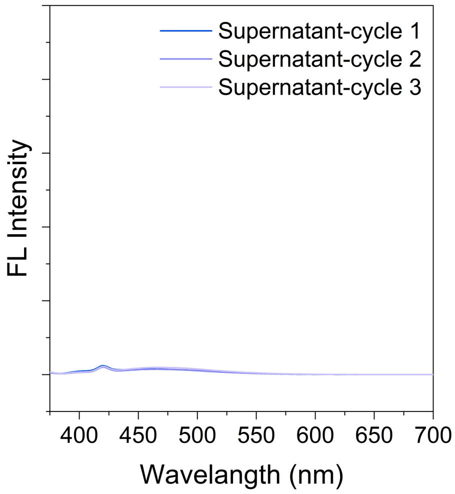

Supplementary Figure 12. Fluorescence spectra of supernatants of TPE-Q18H@GPs during three times centrifugations. λ_{ex}=365 nm.

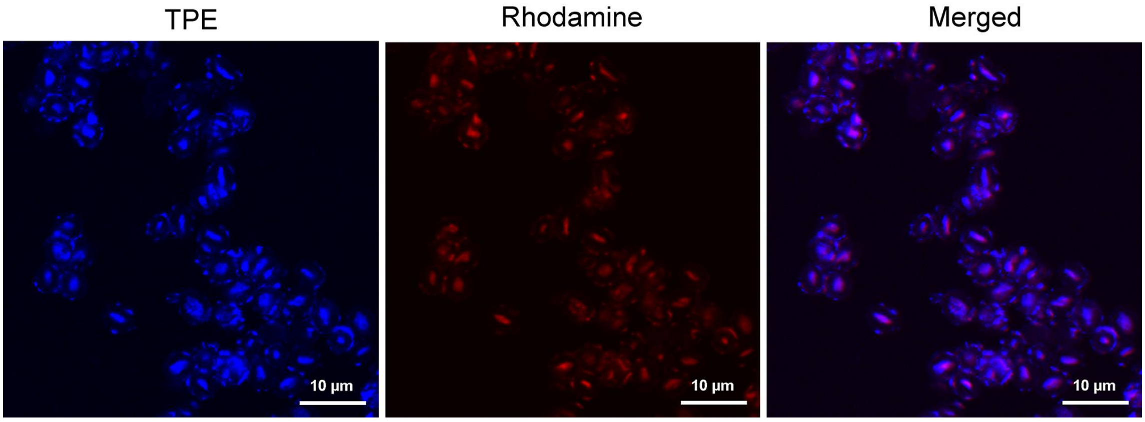

Supplementary Figure 13. Confocal laser scanning microscope images of TPE-Q18H@GPs incubated with Rhodamine. Scale bar: 10 μm.

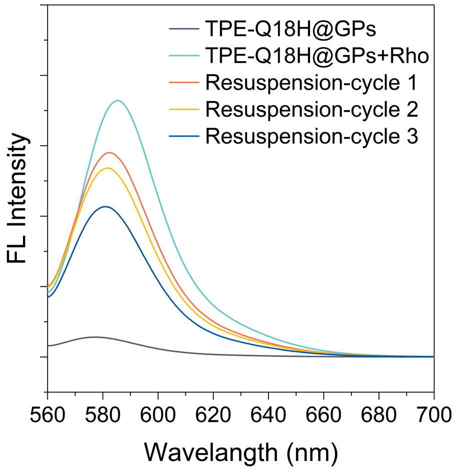

Supplementary Figure 14. Fluorescence spectra of Rhodamine-loaded TPE-Q18H@GPs during three times centrifugations.

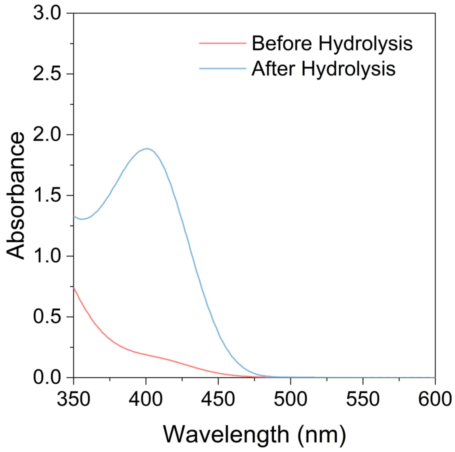

Supplementary Figure 15. UV-vis spectrophotometry of pNPA before and after hydrolysis. Path length= 1.0 mm.

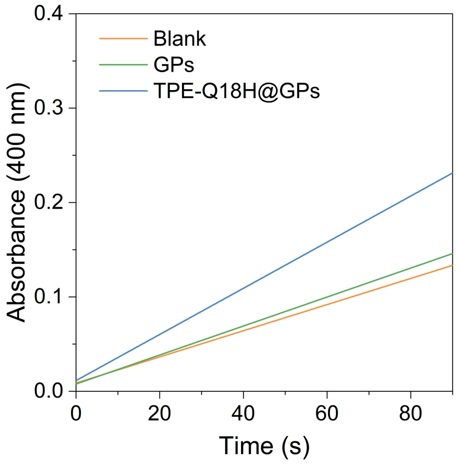

Supplementary Figure 16. Catalytic curves for hydrolysis of pNPA (0.5 mM) in the presence of GPs, and TPE-Q18H@GPs. Path length= 1.0 mm.

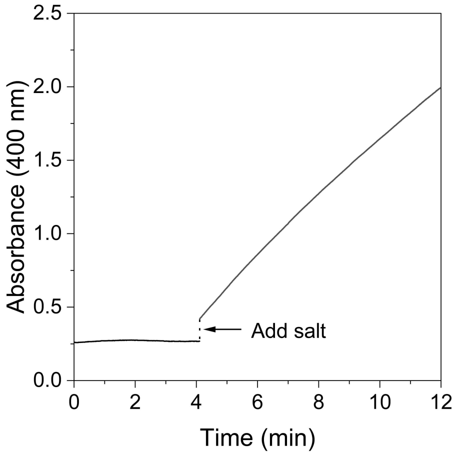

Supplementary Figure 17. Catalytic curves for hydrolysis of pNPA (10 mM) in the presence of TPE-Q18H before and after adding salt. Path length= 10.0 mm.

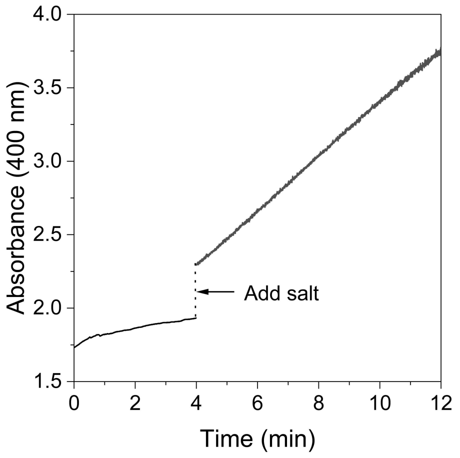

Supplementary Figure 18. Catalytic curves for hydrolysis of pNPA (10 mM) in the presence of TPE-Q18H@GPs before and after adding salt. Path length= 10.0 mm.

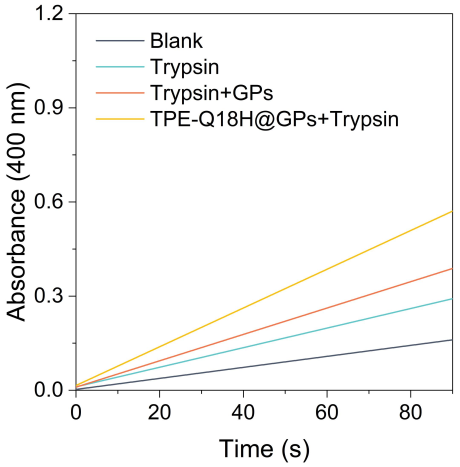

Supplementary Figure 19. Catalytic curves for hydrolysis of pNPA (1 mM) in the presence of trypsin, trypsin with GPs, and trypsin with TPE-Q18H@GPs. Path length= 1.0 mm.
